# ﻿Three new *Dioszegia* species (Bulleribasidiaceae, Tremellales) discovered in the phylloplane in China

**DOI:** 10.3897/mycokeys.101.117174

**Published:** 2024-02-02

**Authors:** Ya-Zhuo Qiao, Shan Liu, Qiu-Hong Niu, Feng-Li Hui

**Affiliations:** 1 School of Life Science and Agricultural Engineering, Nanyang Normal University, Nanyang 473061, China Nanyang Normal University Nanyang China; 2 Research Center of Henan Provincial Agricultural Biomass Resource Engineering and Technology, Nanyang Normal University, Nanyang 473061, China Nanyang Normal University Nanyang China

**Keywords:** Basidiomycota, leaf, phylogenetic analysis, taxonomy, Tremellomycetes

## Abstract

The genus *Dioszegia* is comprised of anamorphic basidiomycetous yeasts and is classified in the family Bulleribasidiaceae of the order Tremellales. Currently, 24 species have been described and accepted as members of the genus, although its diversity and global distribution have not been thoroughly investigated. In this study, yeasts were isolated from plant leaves collected in the Guizhou and Henan Provinces of China and identified through a combination of morphological and molecular methods. Phylogenetic analyses of the combined ITS and LSU sequences coupled with morphological studies revealed three novel species, *D.guizhouensis***sp. nov.**, *D.foliicola***sp. nov.**, and *D.aurantia***sp. nov.**, proposed here. Additionally, our phylogenetic analyses suggest that the recently discovered species *D.terrae* is a synonym of *D.maotaiensis*. This study presents detailed descriptions and illustrations of three new *Dioszegia* species and highlights distinctions between them and their close relatives. The findings of this study contribute to our knowledge of the biodiversity of *Dioszegia*, offering a foundation for future research.

## ﻿Introduction

The genus *Dioszegia* encompasses a group of epiphytic basidiomycetes that inhabit the phylloplane. It was first proposed by Zsolt (1957) based on the single species *Dioszegiahungarica*. Roughly a decade later, the presence of sterigmata or ‘neck-likeconnections’ and lack of ballistoconidia in the species led to its reclassification as a member of the genus *Cryptococcus* (Phaff and Fell 1970). This was later disputed based on new molecular phylogenetic analyses which indicated a great distance between the species and other members of *Cryptococcus* (Takashima and Nakase 1999). In 2001, *Dioszegia* was reinstated and confirmed as a distinct genus based on phylogenetic analysis of the small subunit (SSU) rRNA genes. This finding allowed *D.hungarica* to re-join the genus along with two new combinations, *D.aurantiaca* and *D.crocea* (Takashima et al. 2001). Since then, the genus has expanded and now accommodates a total of 24 described species (Bai et al. 2002; Wang et al. 2003, 2008; Inácio et al. 2005; Connell et al. 2010; Takashima and Nakase 2011; Takashima et al. 2011; Trochine et al. 2017; Li et al. 2020; Maeng et al. 2022). A multi-gene phylogeny placed the genus *Dioszegia* within the newly proposed family Bulleribasidiaceae of the order Tremellales (Liu et al. 2015).

Members of the genus *Dioszegia* share several characteristics that are helpful for phenotypic identification. They exhibit orange or orange-red colonies, polar budding, a non-fermentative nature, and possess co-enzyme Q-10 (Takashima et al. 2001; Takashima and Nakase 2011). Additionally, all known species have thus far only been documented in an asexual stage (Takashima et al. 2001; Wang et al. 2008; Takashima and Nakase 2011). Some species may also form ballistoconidia, hyphae, and poorly developed pseudohyphae (Connell et al. 2010; Li et al. 2020).

Members of *Dioszegia* have been increasingly studied for a wide array of biotechnological applications. The carotenoid-producing abilities of species such as *D.patagonica* and *D.takashimae* offer commercial potential for products such as pigments, nutritional supplements, and pharmaceuticals (Mannazzu et al. 2015). At low temperatures, *D.fristingensis* and *D.patagonica* can secrete extracellular enzymes such as amylase, esterase, pectinase, cellulase, and lipase, making them potential sources of industrially relevant cold-active enzymes (Carrasco et al. 2012; Trochine et al. 2017)

In the past two decades, there has been a flurry of taxonomic research elucidating the diversity of *Dioszegia* species in China. At present, 18 of the 24 accepted *Dioszegia* species have been reported in China, 10 of which were initially described in the country (*D.athyri*, *D.butyracea*, *D.changbaiensis*, *D.heilongjiangensis*, *D.kandeliae*, *D.maotaiensis*, *D.milinica*, *D.ovata*, *D.xingshanensis*, and *D.zsoltii*). The remaining eight species were first documented in other countries (*D.thyrium*, *D.aurantiaca*, *D.butyracea*, *D.cream*, *D.fristingensis*, *D.hungarica*, *D.statzelliae*, *D.takashimae*, and *D.zsoltii*) (Bai et al. 2002; Wang et al. 2003, 2008; Li et al. 2020). There is still much to learn about the *Dioszegia* diversity and distribution in China and beyond. Our recent investigations revealed three new species over two years. This paper aims to employ an integrative taxonomic approach for the delimitation and description of these new taxa, providing a foundation for future investigations of *Dioszegia*.

## ﻿Materials and methods

### ﻿Sample collection and yeast isolation

Leaf samples were collected in the Guiyang Medicinal Botanical Garden (26°53'72"N, 106°70'52"E) and Baotianman Nature Reserve (33°30'44"N, 111°55'47"E) in China. The Guiyang Medicinal Botanical Garden is located in the city of Guiyang in the Yunnan Province of southwest China. With more than 1200 kinds of medicinal plants, it is known as the natural medicine valley. The local climate in this botanical garden is warm winters and fresh and cool summers, with annual mean temperatures around 15.3 °C. The Baotianman Nature Reserve, located in the Henan Province of central China, measures 4,285 ha. With a forest coverage rate of 98%, it is classified as World Biosphere Reserve by the United Nations Educational, Scientific and Cultural Organization (UNESCO). The reserve encompasses a virgin forest with more than 2000 species of vascular plants. The local climate is typical of a transitional climate from northern subtropical zone to warm temperate zone, with cold dry winters, and fresh rainy summers. The annual mean temperature is 15.1 °C.

Yeast strains were isolated from leaf surfaces using the improved ballistospore-fall method as described by Nakase and Takashima (1993). In brief, vaseline was employed to affix fresh and healthy leaves to the inside lids of Petri dishes containing yeast extract-malt extract (YM) agar (0.3% yeast extract, 0.3% malt extract, 0.5% peptone, 1% glucose, and 2% agar). Plates were then incubated at 20 °C until visible colonies had formed. Colonies with different morphotypes were selected and streaked onto additional YM agar plates for purification. After purification, strains were suspended in YM broth supplemented with 20% (v/v) glycerol and stored at −80 °C for future use. All obtained isolates were preserved at the Microbiology Lab, Nanyang Normal University, Henan, China.

### ﻿Morphological and physiological characterization

Phenotypic and physiological characteristics of each yeast isolate were examined using the methods established by Kurtzman et al. (2011). Cell morphology was examined using a Leica DM2500 microscope (Leica Microsystems GmbH, Wetzlar, Germany) equipped with a Leica DFC295 digital microscope color camera under bright field, phase contrast, and differential interference contrast (DIC) conditions. Sexual cycles were investigated for both individual and paired strains on potato dextrose agar (PDA) (20% potato infusion, 2% glucose, and 1.5% agar), corn meal (CM) agar, and yeast carbon base plus 0.01% ammonium sulphate (YCBS) agar for two months and observed at weekly intervals (Li et al. 2020). Ballistoconidium-forming activity was investigated using the inverted-plate method (do Carmo-Sousa and Phaff 1962) after two weeks of incubation on CM agar at 20 °C. Glucose fermentation was observed using Durham fermentation tubes with a liquid medium. Carbon and nitrogen assimilation tests were conducted in a liquid medium, with starved inoculum employed for the latter (Kurtzman et al. 2011). Growth at various temperatures (15, 20, 25, 30, 35, and 37 °C) was determined by cultivation on YM agar. All novel taxonomic descriptions and proposed names were deposited in the MycoBank database (Robert et al. 2013).

### ﻿DNA extraction, PCR amplification, and sequencing

Genomic DNA was extracted from each yeast strain using the Ezup Column Yeast Genomic DNA Purification Kit according to the manufacturer’s instructions (Sangon Biotech Co., Shanghai, China). The ITS region and the D1/D2 domain of the LSU rRNA gene were amplified using primer sets ITS1/ITS4 (White et al. 1990) and NL1/NL4 (Kurtzman and Robnett 1998), respectively. Amplifications were performed in a 25 µL reaction- tube containing 9.5 µL ddH_2_O, 12.5 µL 2× Taq PCR Master Mix with blue dye (Sangon Biotech Co., Shanghai, China), 1 µL DNA template, and 1 µL of each primer. Amplifications were conducted with the following parameters: initial denaturation at 95 °C for 2 min, followed by 35 cycles of 95 ° for C 30 s, 51 °C for 30 s, 72 °C for 40 s, and a final extension at 72 °C for 10 min (Wang et al. 2014). PCR products were purified and sequenced using the same primers by Sangon Biotech Co., Ltd (Shanghai, China). The identity and accuracy of the newly obtained sequences were determined by comparison to GenBank (Sayers et al. 2022) entries. Sequence assembly was conducted using BioEdit v. 7.1.3.0 (Hall 1999). All generated sequences were submitted to GenBank and their corresponding accession numbers are listed in Table 1.

**Table 1.** Taxon names, strain numbers, and GenBank accession numbers used for phylogenetic analyses. Entries in bold were newly generated for this study.

Taxa name	Strain number	GenBank accession numbers	
		ITS	LSU D1/D2
*Bulleribasidiumbegoniae*	CBS 10762^T^	NR_154878	NG_058707
*Bulleribasidiumfoliicola*	CBS 11407^T^	KY101801	NG_058708
*Bulleribasidiumhainanense*	CBS 11409^T^	NR_154879	NG_058709
*Bulleribasidiumoberjochense*	CBS 9110^T^	NR_121467	NG_042388
*Bulleribasidiumpanici*	CBS 9932^T^	NR_121293	NG_058710
*Bulleribasidiumpseudovariabile*	CBS 9609^T^	NR_111085	NG_042393
*Bulleribasidiumsanyaense*	CBS 11408^T^	NR_159742	GQ438831
*Bulleribasidiumsetariae*	CBS 10763^T^	NR_154880	NG_058610
*Bulleribasidiumsiamensis*	CBS 9933^T^	NR_144773	AY188388
*Bulleribasidiumvariabile*	CBS 7347^T^	NR_111058	AF189855
*Bulleribasidiumwuzhishanense*	CBS 11411^T^	NR_153643	GQ438830
***Dioszegiaaurantia* sp. nov.**	**NYNU 229189^T^**	**OP566892**	**OP566893**
*Dioszegiaaurantia* sp. nov.	G.M. 2006-09-03.6 951	OP419710	OP419710
*Dioszegiaantarctica*	CBS 10920 ^T^	NR_159813	FJ640575
*Dioszegiaathyri*	CBS 10119^T^	EU070926	EU070931
*Dioszegiaaurantiaca*	CBS 6980 ^T^	NR_155060	NG_059153
*Dioszegiabuhagiarii*	CBS 10054^T^	NR_073346	NG_059154
*Dioszegiabutyracea*	CBS 10122 ^T^	KY103348	KY107637
*Dioszegiacatarinonii*	CBS 10051^T^	NR_155061	NG_059155
*Dioszegiachangbaiensis*	CBS 9608^T^	NR_136964	NG_059069
*Dioszegiacrocea*	CBS 6714^T^	NR_155062	KY107640
*Dioszegiacryoxerica*	CBS 10919^T^	FJ640565	FJ640562
*Dioszegiadumuzii*	CBS 12501^T^	LT548261	LT548261
***Dioszegiafoliicola* sp. nov.**	**NYUN 229182^T^**	**OP566887**	**OP566888**
***Dioszegiafoliicola* sp. nov.**	**NYNU 229188**	**OP566890**	**OP566889**
***Dioszegiafoliicola* sp. nov.**	**NYNU 2211140**	**OR863956**	**OR863957**
*Dioszegiafristingensis*	CBS 10052 ^T^	NR_136970	NG_070549
***Dioszegiaguizhouensis* sp. nov.**	**NYNU 22985^T^**	**OP566883**	**OP566880**
***Dioszegiaguizhouensis* sp. nov.**	**NYUN 229195**	**OP566896**	**OP581919**
*Dioszegiaheilongjiangensis*	CGMCC 2.5674^T^	NR_174736	MK050291
*Dioszegiahungarica*	CBS 4214^T^	NR_073227	NG_042350
*Dioszegiakandeliae*	CGMCC 2.5658^T^	NR_174739	MK050296
*Dioszegiamaotaiensis*	CGMCC 2.4537^T^	NR_174738	MK050295
*Dioszegiamilinica*	CGMCC2.5628^T^	MK050290	NR_174735
*Dioszegiaovata*	CGMCC 2.3625^T^	NR_174737	MK050294
*Dioszegiapatagonica*	CBS 14901^T^	NR_158412	NG_088008
*Dioszegiarishiriensis*	CBS 11844^T^	NR_157461	NG_059156
*Dioszegiastatzelliae*	CBS 8925^T^	AY029342	AY029341
*Dioszegiatakashimae*	CBS 10053 ^T^	NR_136971	AY562149
*Dioszegiaterrae*	KCTC 27998 ^T^	MZ734406	MZ734403
*Dioszegiaxingshanensis*	CBS 10120^T^	KY103359	KY107649
*Dioszegiazsoltii var. yunnanensis*	CBS 9128^T^	NR_156190	NG_070550
*Dioszegiazsoltiivar.zsoltii*	CBS 9127^T^	AF385445	NG_059157
*Nielozymaformosana*	CBS 10306 ^T^	NR_154221	NG_058356
*Nielozymamelastomae*	CBS 10305^T^	NR_154221	AB119464
*Sugitazymamiyagiana*	CBS 7526^T^	NR_073237	AF189858

CBS, CBS-KNAW Collections, Westerdijk Fungal Biodiversity Institute, Utrecht, The Netherlands; CGMCC, China General Microbiological Culture Collection Center, Beijing, China; KCTC, Korea Collection for Type Cultures, KRIBB, Korea; NYNU, Microbiology Lab, Nanyang Normal University, Henan, China; **^T,^** type strain. Species obtained in this study are in bold.

### ﻿Phylogenetic analysis

Phylogenetic analyses employed a total of 92 nucleotide sequences, including 12 novel sequences generated in this study. The remaining sequences were obtained from previous studies (Li et al. 2020; Maeng et al. 2022) and GenBank (Table 1). *Sugitazymamiyagiana* CBS 7526^T^ was used as the outgroup. Phylogenetic relationships between the new *Dioszegia* species and their close relatives were determined using a combined ITS and LSU sequence dataset. Sequences of individual markers were aligned with either Clustal X v. 1.83 (Thompson et al. 1997) or MAFFT v. 7.110 (Katoh and Standley 2013) using default settings. Aligned sequences of the different markers were concatenated with PhyloSuite v. 1.2.2 (Zhang et al. 2020). Alignments were improved through manual gap adjustments. Ambiguously aligned regions were excluded prior to analysis.

Phylogenetic analyses were conducted employing both maximum likelihood (ML) and Bayesian inference (BI). ML was determined with 1,000 searches on RAxML v. 8.2.3 (Stamatakis 2014) and ML bootstrap values (MLBS) were assessed through 1,000 rapid bootstrap replicates using the GTRCAT model. For BI, ModelFinder (Kalyaanamoorthy et al. 2017) was used to determine the optimal substitution model to fit the DNA evolution. BI data was analysed with MrBayes v. 3.2.7a (Ronquist et al. 2012) through the CIPRES Science Gateway version 3.3. Best-fit evolution models for the ITS and LSU partitions were GTR+I+G. Six simultaneous Markov chains were run for 50 million generations with trees being sampled every 1,000^th^ generation. The first 25% of created sample trees were discarded as the burn-in phase of analysis. The remaining trees were used to infer Bayesian posterior probabilities (BPP) for the clades.

The resulting trees were viewed in FigTree v. 1.4.3 (Andrew 2016) and processed with Adobe Illustrator CS5. Branches that received MLBS ≥ 50% and BPP ≥ 0.95 were considered significantly supported.

## ﻿Results

### ﻿Molecular phylogeny

**Figure 1.**
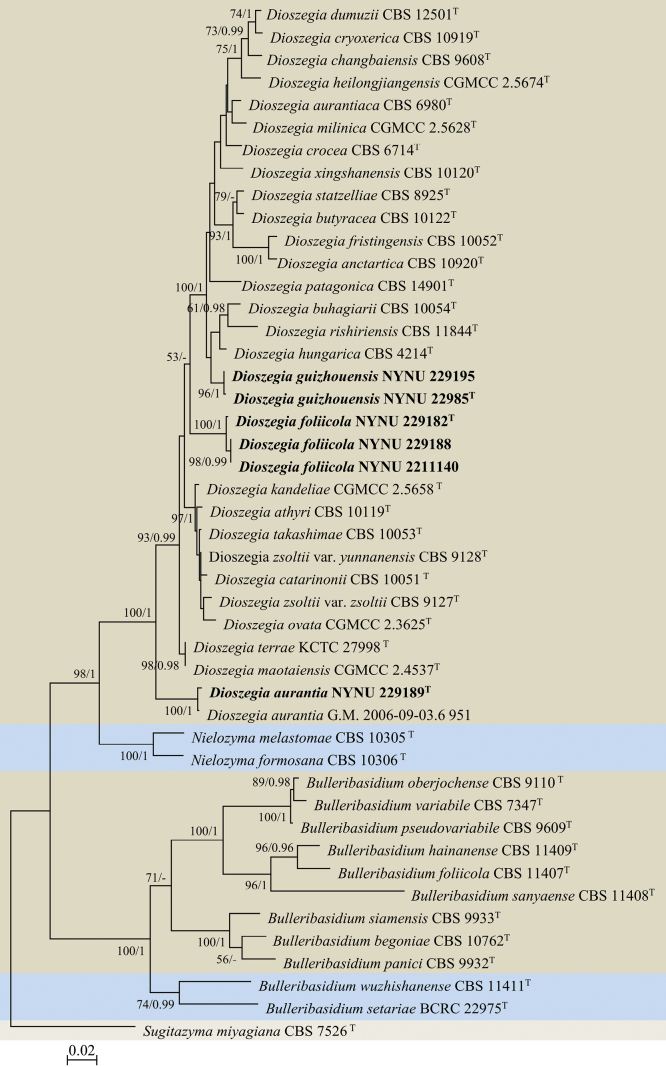
Maximum likelihood (ML) phylogram of *Dioszegia* species and close relatives based on combined ITS and LSU sequence data. *Sugitazymamiyagiana* CBS 7526^T^ serves as the outgroup. Branches are labelled with MLBS ≥ 50% and BPP ≥ 0.95. Novel strains are highlighted in bold.

This study presents the discovery of three novel *Dioszegia* species represented by six strains isolated from leaf samples in the provinces of Guizhou and Henan (Table 2). The combined ITS and LSU sequence data was utilized to elucidate the phylogenetic positions of the new species. 120 aligned positions were excluded from the alignment due to problematic homology assessment. This final dataset consisted of 997 characters, 588 from ITS and 409 from LSU. Among these, 604 were constant and 393 were variable, out of which 292 were parsimony-informative. Finally, 101 were singletons. The topology of the ML and Bayesian trees was consistent with each other, and only the ML tree is shown (Fig. 1). The five strains isolated in this study formed three strongly supported groups (100% MLBS/1 BPP), distinct from other known species of *Dioszegia*.

**Table 2.** Strains representing the novel species described in this study and relevant information associated to them.

Strain	Source	Location
*Dioszegiaguizhouensis* sp. nov.		
NYNU 22985^T^	Leaf of *Schisandra* sp.	Guiyang Medicinal Botanical Garden, Guiyang, Guizhou Province, China
NYUN 229195	Leaf of *Mussaendae* sp.	Guiyang Medicinal Botanical Garden, Guiyang, Guizhou Province, China
*Dioszegiafoliicola* sp. nov.		
NYUN 229182^T^	Leaf of *Salvia* sp.	Guiyang Medicinal Botanical Garden, Guiyang, Guizhou Province, China
NYNU 229188	Leaf of *Broussonetiapapyrifera*	Guiyang Medicinal Botanical Garden, Guiyang, Guizhou Province, China
NYNU 2211140	Leaf from an unidentified tree	Baotianman Nature Reserve, Nanyang, Henan Province, China
*Dioszegiaaurantia* sp. nov.		
NYNU 229189^T^	Leaf of *Cornusofficinalis*	Guiyang Medicinal Botanical Garden, Guiyang, Guizhou Province, China

The strains NYUN 22985 and NYUN 229195 had similar sequences with only one nt difference in the ITS region, suggesting that they belong to the same species. Two strains in the NYUN 22985 group formed a separate branch on the phylogenetic tree (Fig. 1), forming a clade with *D.hungarica*, the *Dioszegia* type species, and 15 other known species with strong support (100 MLBS/1 BPP). BLASTn searches of the D1/D2 and ITS sequences indicated that *D.hungarica* is the closet relative, differing by four nt (~0.7%) substitutions in the D1D2 domain and 14–15 nt (~2.9–3.1%) mismatches in the ITS region. The NYUN 22985 group is considered a distinct *Dioszegia* species based on the basidiomycetous yeast species threshold (Fell et al. 2000; Vu et al. 2016), which suggests that strains differing by two or more nucleotide substitutions in the D1/D2 domains or exhibiting 1–2% nucleotide differences in the ITS regions may represent different taxa. Therefore, *D.guizhouensis* sp. nov. is proposed as a novel *Dioszegia* species to accommodate the strains.

Three strains, viz. NYNU 229182, NYNU 229188, and 2211140, possessed mutually similar sequences with three nt differences in the D1/D2 region and one in the ITS region, indicating conspecificity. Additionally, the NYNU 229182 group shared similar D1/D2 sequences (one to two nt differences) with the GenBank isolate WOct07D (2)-Y3 (GQ352531) identified as ‘*Dioszegiazsoltii*’, suggesting another conspecific relationship. BLASTn searches of the D1/D2 sequences indicated that this group was most closely related to *D.maotaiensis* and *D.terrae*, differing by 10–11 nt (~1.7–1.8%) substitutions in the D1/D2 domain and more than 27 nt (5.4%) mismatches in ITS region. Thus, the group represents a novel *Dioszegia* species, for which the name *D.foliicola* sp. nov. is proposed.

Strain NYNU 229189 grouped with G.M.2006-09-03.6951 (OP419710), an unpublished strain obtained from the bark of rotting branches collected in Australia, which jointly were placed as a separate branch as the sister clade to the remaining part of of *Dioszegia* (Fig. 1). The two strains differed by only two and four nt differences in the D1/D2 and ITS region, respectively, suggesting conspecificity. NYNU 229189 is closely related to *D.maotaiensis* and *D.terrae*, differing from the latter two by 16 nt (~2.7%) substitutions in the D1/D2 domain and more than 23 nt (~5.7%) mismatches in the ITS region. This suggests that NYNU 229189 represents a new *Dioszegia* species, for which the name *D.aurantia* sp. nov. is proposed.

### ﻿Taxonomy

#### ﻿ Dioszegia guizhouensis

Taxon classificationFungiTremellalesBulleribasidiaceae

Y.Z. Qiao & F.L. Hui sp. nov.

90FDAA78-117A-5B83-B912-AFE89E74F489

MycoBank No: 851291

Fig. 2A

##### Etymology.

The specific epithet *guizhouensis* refers to the geographic origin of the type strain, Guizhou province.

##### Typus.

China, Guizhou Province, Guiyang City, Guiyang Botanical Garden, in the phylloplane of *Schisandra* sp., September 2022, L. Zhang and F.L. Hui, NYUN 22985 (holotype GDMCC 2.311^T^ preserved as a metabolically inactive state, culture ex-type PYCC 9938).

**Figure 2.**
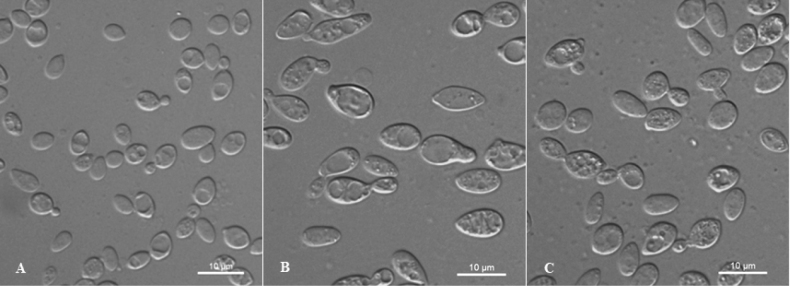
Vegetative cells of *Dioszegiaguizhouensis* sp. nov. NYNU 22985^T^ (**A**), *Dioszegiafoliicola* sp. nov. NYUN 229182^T^ (**B**), and *Dioszegiaaurantia* sp. nov. NYNU 229189^T^ (**C**) following growth in YM broth for 7 days at 20 °C. Scale bars: 10 μm.

##### Description.

On YM agar, after 7 days at 20 °C, the streak culture is pink to orange, butyrous, smooth. The margin is entire. On YM agar, after 7 days at 20 °C, cells are ovoid and ellipsoidal, 2.8–4.6 × 4.1–6.8 μm and single, budding is polar. After 1 month at 20 °C, a ring and sediment are present. In Dalmau plate culture on corn meal agar, hyphae and pseudohyphae are not formed. Sexual structures are not observed for individual strains and strain pairs on PDA, CM agar, and YCBS agar for two months. Ballistoconidia are not produced on CM agar after two weeks at 20 °C. Glucose fermentation is absent. Glucose, sucrose, raffinose, melibiose, galactose, trehalose, maltose, melezitose, cellobiose, salicin, L-sorbose (delayed), L-rhamnose, D-xylose, L-arabinose, D-arabinose, 5-keto-D-gluconate (weak), D-ribose, galactitol, D-mannitol, D-glucitol, succinate (weak), citrate, D-gluconate, N-acetyl-D-glucosamine, 2-keto-D-gluconate, D-glucuronate, and glucono-1,5-lactone are assimilated as carbon sources. Inulin, lactose, methyl-α-D-glucoside, methanol, ethanol, glycerol, erythritol, ribitol, myo-inositol, DL-lactate, and D-glucosamine are not assimilated. Nitrite is assimilated as the sole nitrogen source. Nitrate, ethylamine, L-lysine, and cadaverine are not assimilated. Maximum growth temperature is 30 °C. Growth in vitamin-free medium is positive. Starch-like substances are produced. Urease activity is positive. Diazonium Blue B reaction is positive.

##### Additional strain examined.

China, Guizhou Province, Guiyang City, Guiyang Botanical Garden, in the phylloplane of *Mussaendae* sp., September 2022, L. Zhang and F.L. Hui, NYUN 229195.

##### GenBank accession numbers.

Holotype NYUN 22985^T^ (ITS: OP566883, D1/D2: OP566880); additional strain 229195 (ITS: OP566896, D1/D2: OP581919).

##### Note.

*Dioszegiaguizhouensis* sp. nov. can be physiologically differentiated from its closest known species *D.hungarica* (Takashima and Nakase 2011) by its inability to assimilate D-glucosamine, its ability to assimilate melibiose and L-sorbose, and its capacity to grow in vitamin-free medium and at 30 °C.

#### ﻿ Dioszegia foliicola

Taxon classificationFungiTremellalesBulleribasidiaceae

Y.Z. Qiao & F.L. Hui sp. nov.

25F39E25-7065-5997-963F-C3C25D79CD1E

MycoBank No: 851294

Fig. 2B

##### Etymology.

The specific epithet *foliicola* refers to the type strain isolated from a leaf.

##### Typus.

China, Guizhou Province, Guiyang City, Guiyang Botanical Garden, in the phylloplane of *Salvia* sp., September 2022, L. Zhang and F.L. Hui, NYUN 229182 (holotype GDMCC 2.316^T^ preserved as a metabolically inactive state, culture ex-type PYCC 9939 and CICC 33571).

##### Description.

On YM agar, after 7 days at 20 °C, the streak culture is orange, butyrous, smooth. The margin is entire. On YM agar, after 7 days at 20 °C, cells are ovoid and ellipsoidal, 3.9–4.8 × 4.8–7.9 μm and single, budding is polar. After 1 month at 20 °C, a ring and sediment are present. In Dalmau plate culture on corn meal agar, hyphae and pseudohyphae are not formed. Sexual structures are not observed for individual strains and strain pairs on PDA, CM agar and YCBS agar for two months. Ballistoconidia are not produced on CM agar after two weeks at 20 °C. Glucose fermentation is absent. Glucose, sucrose, raffinose, melibiose, galactose, trehalose, maltose, melezitose, methyl-α-D-glucoside, cellobiose, salicin, L-sorbose, L-rhamnose, D-xylose, L-arabinose, D-arabinose, 5-keto-D-gluconate, D-ribose, galactitol, D-mannitol, succinate, D-gluconate, N-acetyl-D-glucosamine, 2-keto-D-gluconate and D-glucuronate are assimilated as carbon sources. Inulin, lactose, methanol, ethanol, glycerol, erythritol, ribitol, D-glucitol, myo-inositol, DL-lactate, citrate, D-glucosamine, and glucono-1,5-lactone are not assimilated. Nitrite and L-lysine are assimilated as nitrogen sources. Nitrate, ethylamine, and cadaverine are not assimilated. Maximum growth temperature is 30 °C. Growth in vitamin-free medium is positive. Starch-like substances are produced. Urease activity is positive. Diazonium Blue B reaction is positive.

##### Additional strain examined.

China, Guizhou Province, Guiyang City, Guiyang Botanical Garden, in the phylloplane of *Broussonetiapapyrifera*, September 2022, L. Zhang and F.L. Hui, NYUN 229188 and China, Henan Province, Nanyang City, Baotianman Nature Reserve, in the phylloplane from an unidentified tree, October 2022, J.Z. Li, NYUN 2211140.

##### GenBank accession numbers.

Holotype GDMCC 2.316^T^ (ITS: OP566887, D1/D2: OP566888); additional strains NYUN 229188 (ITS: OP566890, D1/D2: OP566889) and NYUN 2211140 (ITS: OR863956, D1/D2: OR863957).

##### Note.

*Dioszegiafoliicola* sp. nov. can be physiologically differentiated from its closest known species *D.maotaiensis* (Li et al. 2020) by its inability to assimilate inulin and citrate, its ability to assimilate methyl-α-D-glucoside, salicin, L-sorbose, D-ribose, galactitol, and D-mannitol, and its capacity to grow at 30 °C.

#### ﻿ Dioszegia aurantia

Taxon classificationFungiTremellalesBulleribasidiaceae

Y.Z. Qiao & F.L. Hui sp. nov.

0131EA33-0027-5A9B-85C7-668E05EE7D4A

MycoBank No: 851296

Fig. 2C

##### Etymology.

The specific epithet *aurantia* refers to the *aurantiaca* colony morphology.

##### Typus.

China, Guizhou Province, Guiyang City, Guiyang Botanical Garden, in the phylloplane of *Cornusofficinalis*, September 2022, L. Zhang and F.L. Hui, NYUN 229189 (holotype GDMCC 2.335^T^ preserved as a metabolically inactive state, culture ex-type PYCC 9937 and CICC 33572).

##### Description.

On YM agar, after 7 days at 20 °C, the streak culture is orange, butyrous, smooth. The margin is entire. On YM agar, after 7 days at 20 °C, cells are ovoid and ellipsoidal, 4.6–5.0 × 5.0–8.2 μm and single, budding is polar. After 1 month at 20 °C, a ring and sediment are present. In Dalmau plate culture on corn meal agar, hyphae and pseudohyphae are not formed. Sexual structures are not observed for individual strains and strain pairs on PDA, CM agar, and YCBS agar for two months. Ballistoconidia are not produced on CM agar after two weeks at 20 °C. Glucose fermentation is absent. Glucose, inulin, sucrose, raffinose, melibiose, galactose, trehalose, maltose, melezitose, methyl-α-D-glucoside (delayed), cellobiose, salicin (weak), L-sorbose (delayed), L-rhamnose (delayed and weak), D-xylose, L-arabinose, D-arabinose (weak), 5-keto-D-gluconate, D-ribose, galactitol, D-mannitol, D-glucitol, succinate (weak), N-acetyl-D-glucosamine, 2-keto-D-gluconate (delayed and weak), and D-glucuronate are assimilated as carbon sources. Lactose, methanol, ethanol, glycerol, erythritol, ribitol, myo-inositol, DL-lactate, citrate, D-gluconate, D-glucosamine, and glucono-1,5-lactone are not assimilated. Nitrite (delayed) and L-lysine (delayed and weak) are assimilated as nitrogen sources. Nitrate, ethylamine, and cadaverine are not assimilated. Maximum growth temperature is 25 °C. Growth in vitamin-free medium is negative. Starch-like substances are produced. Urease activity is positive. Diazonium Blue B reaction is positive.

##### GenBank accession numbers.

Holotype GDMCC 2.335^T^ (ITS: OP566892, D1/D2: OP566893).

##### Note.

*Dioszegiaaurantia* sp. nov. can be physiologically differentiated from its closest known species *D.maotaiensis* (Li et al. 2020) by its inability to assimilate citrate, its ability to assimilate methyl-α-D-glucoside, salicin, L-sorbose, D-ribose, D-mannitol, D-glucitol, and N-acetyl-D-glucosamine, and its capacity to grow in vitamin-free medium and at 30 °C.

## ﻿Discussion

In this study, we present three novel *Dioszegia* species discovered in China: *D.guizhouensis* sp. nov., *D.foliicola* sp. nov., and *D.aurantia* sp. nov. This work provides a comprehensive description of each species based on molecular analyses and morphological examinations. Moreover, our phylogenetic analyses illustrate clear distinctions between each new species and other members of *Dioszegia*, which was confirmed as a monophyletic genus in a strongly supported clade (Fig. 1). Pairwise sequence comparisons of the D1/D2 domain and the ITS region of the novel species and their close relatives support species differentiation based on the common threshold applied to basidiomycetous yeasts (Fell et al. 2000; Vu et al. 2016). The new species were highly similar in cell shape, colony morphology, and color, but differed from closely related species in terms of physiological and biochemical characteristics. Therefore, the results of our molecular phylogenetic analyses and phenotypic examinations support the description of three new *Dioszegia* species.

Several new species have been added to *Dioszegia* recently (Li et al. 2020; Maeng et al. 2022). Notably, our phylogenetic analyses revealed that the recently described species *D.terrae* clustered with *D.maotaiensis* in a well-supported clade within *Dioszegia* (Fig. 1). *D.maotaiensis* was described first and the description of *D.terrae* seminly overlooked the previously validly described species *D.maotaiensis*. These two species had only one nt difference in the ITS region, suggesting that *D.terrae* is a synonym of *D.maotaiensis*. Consequently, 26 species, including three new species described in the present study, are currently included in the genus *Dioszegia*.

Members of the genus *Dioszegia* are widely distributed across a variety of habitats. Although isolates are commonly obtained as epiphytic phylloplane fungi in temperate and subtropical climate regions (Inácio et al. 2005; Wang et al. 2008; Li et al. 2020), previous studies have also collected samples from roots (Renker et al. 2004) and soil (Takashima et al. 2011; Maeng et al. 2022). Additionally, isolates have also been collected from cold substrates such as snow (Trochine et al. 2017), glacial melt (de García et al. 2007; Trochine et al. 2017), and polar desert soil (Connell et al. 2010). In this study, six strains of three new *Dioszegia* species share with most other species in the genus association with plant leaves. The results further confirm that the natural distribution of *Dioszegia* species in the phylloplane is common. Furthermore, strain WOct07D (2)-Y3 (GQ352531), identified as ‘*Dioszegiazsoltii*’ from USA, is conspecific with *D.foliicola* sp. nov., while strain G.M.2006-09-03.6951 (OP419710) from Australia is conspecific with *D.aurantia* sp. nov. These observations suggests that the two new species *D.foliicola* sp. nov. and *D.aurantia* sp. nov. may be broadly distributed outside of China. Indeed, further large-scale studies are needed to explore the diversity and distribution of *Dioszegia* species worldwide. *D.fristingensis* is a versatile extremophilic species that has been frequently found in plants inhabiting hyper-arid, alkaline, and hypersaline environments (Abu-Ghosh et al. 2014; Wei et al. 2022), implying that this species may help plants survive in dry areas. We also isolated six strains of three novel *Dioszegia* species—*D.guizhouensis* sp. nov., *D.foliicola* sp. nov., and *D.aurantia* sp. nov.—from plant leaves, and it is possible that these species provide similar ecological functions benefits to their hosts as does *D.fristingensis*.

Many *Dioszegia* species have adapted to tolerate challenges presented by their environments. Notably, more than 10 *Dioszegia* species are known to accumulate mycosporin-glutamine-glucoside (MGG), a UVB-absorbing molecule that acts in response to photostimulation (Trochine et al. 2017). *D.patagonica* even contains higher levels of MGG than *Phaffiarhodozyma*, which is recognized for its ability to endure UV-B radiation (Madhour et al. 2005; Libkind et al. 2009). Further exploration of *Dioszegia* diversity is necessary to determine whether MGG is associated with other taxonomic traits or influences UV radiation tolerance (Libkind et al. 2009).

## Supplementary Material

XML Treatment for Dioszegia guizhouensis

XML Treatment for Dioszegia foliicola

XML Treatment for Dioszegia aurantia

## ﻿Additional information

### Author ORCIDs

Ya-Zhuo Qiao https://orcid.org/0009-0000-9074-2443

Shan Liu https://orcid.org/0009-0003-2845-1495

Qiu-Hong Niu https://orcid.org/0000-0003-1695-7117

Feng-Li Hui https://orcid.org/0000-0001-7928-3055

### Data availability

All of the data that support the findings of this study are available in the main text or Supplementary Information.

## Supplementary materials

Supplementary material 1 Molecular data Data type fas Explanation note A dataset of ITS and LSU for Fig. 1. https://binary.pensoft.net/file/978060This dataset is made available under the Open Database License (http://opendatacommons.org/licenses/odbl/1.0/). The Open Database License (ODbL) is a license agreement intended to allow users to freely share, modify, and use this Dataset while maintaining this same freedom for others, provided that the original source and author(s) are credited.Ya-Zhuo Qiao, Shan Liu, Qiu-Hong Niu, Feng-Li Hui
